# HRG C633T polymorphism and risk of gestational hypertensive disorders: a pilot study

**DOI:** 10.1186/s12881-018-0550-8

**Published:** 2018-03-14

**Authors:** Evangelia Elenis, Alkistis Skalkidou, Agneta Skoog-Svanberg, Gunilla Sydsjö, Anneli Stavreus-Evers, Helena Åkerud

**Affiliations:** 1 Department of Women’s and Children’s Health, Uppsala University, -751 85 Uppsala, SE Sweden; 2 Obstetrics and gynaecology, Department of Clinical and Experimental Medicine, Faculty of Health Sciences, Linköping University, Linköping, Sweden

**Keywords:** Angiogenesis, Gestational hypertensive disorders, HRG, HRG C633T SNP, Preeclampsia

## Abstract

**Background:**

Preeclampsia and gestational hypertensive disorders are thought to occur due to endothelial cell dysfunction and abnormal placentation, triggered by angiogenesis-related factors yet undetermined. The aim of this study was to investigate whether a genetic polymorphism (SNP) of Histidine-rich glycoprotein (HRG), HRG C633T SNP, is associated with gestational hypertensive disorders.

**Methods:**

It was performed a nested case-control study from the BASIC Cohort of Uppsala University Hospital comprising 92 women diagnosed with gestational hypertensive disorders without other comorbidities and 200 women with full term uncomplicated pregnancies, all genotyped regarding HRG C633T SNP.

**Results:**

The genetic analysis of the study sample showed that C/C genotype was more prevalent among controls. The presence of the T-allele showed a tendency towards an increased risk of gestational hypertensive disorders. After clustering the study participants based on their genotype, it was observed that the odds for gestational hypertensive disorders among heterozygous C/T or homozygous T/T carriers were higher compared to homozygous C/C carriers [OR 1.72, 95% CI (1.04–2.84)]. The association remained significant even after adjustment for maternal age, BMI and parity.

**Conclusions:**

The HRG C633T genotype seems to be associated with gestational hypertensive disorders, and as part of a greater algorithm, might contribute in the future to the prediction of the individual susceptibility to the condition.

## Background

Gestational hypertensive disorders (i.e. gestational hypertension, preeclampsia and eclampsia, and preeclampsia superimposed on chronic hypertension) are syndromes of systemic vascular endothelial dysfunction that are encountered after the 20th week of gestation and can present as late as six weeks after delivery [1]. Among hypertensive disorders, preeclampsia is estimated to affect 2–8% of pregnancies globally and accounts for 10–15% of maternal mortality, as well as for 25% of peri- and neo-natal mortality worldwide [2].

It is nowadays becoming a widely accepted hypothesis that dysregulation of placental vasculature underlies an array of pregnancy complications such as preeclampsia, intrauterine growth restriction and probably recurrent miscarriage, suggesting that they all represent different representations of the same placental-mediated pathology [3] due to inadequate angiogenesis [4]. Despite still being a subject of debate, recent theories on the pathogenesis of preeclampsia focus on placental damage already taking place early in pregnancy. It has been proposed that maternal immunological intolerance to the fetus results in altered trophoblast function. The inappropriate immune response that underlies the shallow trophoblast invasion leads to deficient remodelling of the maternal spiral arteries that perfuse the placenta. The latter results in maternal systemic disease through primary mediators, including oxidative stress and inflammation, and also secondary mediators, including modifiers of endothelial function and angiogenesis [5], leading to the clinical manifestation of preeclampsia. Genetic studies, such as microarray-based gene expression profiling studies, have been employed in the investigation of the pathogenesis of preeclampsia and differentially expressed genes have been identified [6, 7].

The Histidine-rich glycoprotein (HRG) is a multi-domain plasma protein produced by parenchymal liver cells and transported either as a free protein or stored in *a*-granules of platelets and released after thrombin stimulation [8]. Its exact role still remains obscure but it has been shown to be involved in the immune system, in coagulation, as well as in angiogenesis and in fertility regulation. Supporting the latter biologic function, is the abundance of HRG throughout the female reproductive tract (i.e. in follicular fluid, endometrium, fallopian tube and in myometrium) where the oocyte develops, is fertilized and later implants [9]. Regarding its function, HRG seems to act as an adaptor molecule, exhibiting both pro- and anti-angiogenic properties, depending on the components of the microenvironment or on proteolytic cleavage of the antiangiogenic fragment of HRG (i.e. the HRR fragment) [10]. More specifically, it has been previously shown that HRG is involved in the hypercoagulability and the angiogenic imbalance seen in early-onset preeclampsia [11, 12]. Furthermore, specific HRG polymorphisms have been associated with recurrent miscarriage [13, 14] and infertility [9] with the possible hypothesis that the amino acid substitution induces a conformational change on the HRG protein which affects its function [15]. It has thus been further hypothesized that HRG, by regulating angiogenesis, might be an important intermediary for adequate implantation and placentation [16]*.* Nonetheless, gene expression studies, validated by proteomics and metabolomics, are required in order to fully understand the underlying regulatory pathways involved in fertility and pregnancy related disorders.

The *HRG* gene is located at chromosome 3 and contains seven exons and six introns. The HRG protein has been reported to exist as 10 naturally occurring isoforms within the gene corresponding to single nucleotide polymorphisms (SNP). In one of them, a cytosine nucleotide (C) is replaced by a thymidine (T) at position 633 in the mRNA sequence denoted as C633T (also known as rs 9898 C/T) which results in an amino acid change from proline to serine at position 204 in the protein (in some publications this amino acid is denoted as occupying position 186 in the protein corresponding to the molecule without its signal peptide). The isoform of HRG-containing proline has a molecular weight of 75 kDa, while the corresponding serine-containing isoform weighs 77 kDa due to an extra glycosylation site at amino acid position 202 [15]*.* It has been described that the tridimensional structure of the entire HRG molecule is stabilized by six disulphide bonds that protect the molecule from dispersing upon proteolytic digestion [8]. However, the redox modification of one disulphide bond (Cys 185–Cys 407), entailing the HRR region, together with plasmin cleavage of HRG, releases the HRR fragment, which is the only fragment with antiangiogenic potential. Therefore, the critical topographic position of the HRG C633T polymorphism in the vicinity of that susceptible disulphide bond, renders it as an interesting study focus, as already seen even in other fertility related processes [9, 14, 17].

In summary, an association between serum levels of the HRG protein during pregnancy and the development of preeclampsia, as well as an association between HRG C633T genetic polymorphism and recurrent miscarriage, have been noted. Prompted by the latter, we aimed to investigate whether gestational hypertensive disorders are associated with the HRG C633T SNP in women pregnant with singletons without previous comorbidities associated with hypertension.

## Methods

### Study design

The study was designed as a nested case-control study conducted as a part of the cohort project, “Biology, Affect, Stress, Imaging and Cognition in pregnancy and the puerperium” (BASIC). The BASIC project is a longitudinal study investigating biological correlates of mood and anxiety disorders during pregnancy and in the postpartum period. The groups of cases and controls were recruited from the Department of Obstetrics and Gynecology at Uppsala University Hospital, Sweden between the years 2009 and 2015. Within the context of the BASIC-study, all pregnant women in Uppsala county who were older than 18 years of age and could adequately communicate in Swedish were invited to participate while attending their routine ultrasound in gestational weeks 16–18. Participation rate was estimated at 22% [18]. Following the obtaining of oral and written informed consent, a plasma sample was collected. Participants of this study were followed up until delivery and relevant information on obstetric and perinatal variables, health problems and medication were retrieved after scrutinization of their medical records.

### Study population

Only women who delivered singletons without pathologic pregnancies as diagnosed by routine ultrasound were included in this sub-study. Women with known risk factors for preeclampsia and hypertension, such as systemic lupus erythematosus (SLE), diabetes mellitus, known vascular disease, severe thrombophilia and renal disease, were excluded. Control participants were randomly chosen from the BASIC cohort and were defined as healthy spontaneously pregnant women with singletons, without being diagnosed with hypertensive disorders of pregnancy or any of the comorbidities mentioned above. Eligible cases with a diagnosis of new onset and superimposed preeclampsia, eclampsia, gestational hypertension and HELLP (i.e., a syndrome with three main features such as hemolysis, elevated liver enzymes and low platelets) were identified by reviewing their medical records after delivery. Because the inclusion of the study participants was initiated before 2013, the diagnostic definition of preeclampsia used in the identification of cases is reflective of the older definition. Gestational hypertension was defined as the presence of hypertension (BP ≥140/90 mmHg) on at least 2 occasions after the 20th gestational week in a woman with previously normal blood pressure. Preeclampsia was defined as the presence of hypertension (BP ≥140/90 mmHg) after 20 weeks’ gestation in connection with proteinuria (≥ 0.3 g protein in 24-h urine specimen) [19].

### Study parameters

The following study parameters were evaluated: maternal age at delivery for the gestation during study inclusion (completed years upon delivery); maternal BMI (kg/m^2^) estimated at first antenatal visit at prenatal center (week 10 of gestation); nicotine use (cigarettes/snus) during pregnancy; parity (primi- or multiparity); genotypic distribution regarding the HRG C633T SNP (homozygous C/C, heterozygous C/T and homozygous T/T carriers); neonatal gender (male/female); gestational length upon delivery estimated by ultrasound during the second trimester or last menstrual period if no ultrasound was performed; birth weight (grams); and length (cm) of the infant.

### Blood sample collection

Blood samples were collected after study inclusion in EDTA-containing tubes and centrifuged at 1500 *g* for 10 min. Plasma and buffy coat were separated, transferred to new tubes and stored at − 70 °C until analysis.

### DNA extraction and SNP analysis

With the use of the QIAamp DNA Blood Maxi kit (Qiagen, Venlo, the Netherlands) we extracted genomic DNA from the buffy coat. The extraction was performed according to the standard protocol. The samples were genotyped for the HRG C633T SNP (rs 9898), using the TaqMan SNP Genotyping Assay (Applied Biosystems, Foster City, CA, USA). Briefly, PCR was performed in a 96-well plate in a total volume of 25 μl for each well. Each reaction consisted of 1 x TaqMan Universal PCR Master Mix (PCR buffer, ROX passive reference dye, dNTPs and AmpliTaq Gold polymerase), 1 x SNP Genotyping Assay (sequence-specific forward and reverse primers to amplify the polymorphic sequence of interest, i.e. HRG exon 5, TaqMan MGB probes labelled with VIC dye to detect allele 1 sequence and with FAM to detect allele 2 sequence) and 10 ng of genomic DNA. Cycling conditions were initiated for 10 min at 95 °C followed by 40 cycles of 15 s at 92 °C and 1 min at 60 °C. Real-time fluorescence detection was carried out. Sequence Detection System software (Applied Biosystems) was used to plot fluorescence (Rn) values based on the signals from each well. The plotted fluorescence signals indicated which alleles were present in each sample.

### Statistical analyses

All statistical analyses were performed using IBM SPSS Statistics 20.0 software (IBM Corporation, Armonk, NY, USA). The normality of the distribution of continuous variables was determined with the Shapiro-Wilk test. Because most continuous variables were not normally distributed (with the exception of maternal age and birthweight), baseline characteristics were presented as medians and interquartile range. Non-parametric tests, such as the Mann-Whitney *U* test and the Kruskal-Wallis test were performed for the comparison of non-normally distributed variables. For the comparison of categorical variables, the Chi square test was conducted. Demographic and clinical data were compared among cases and controls and genotype groups. The number of the homozygous and heterozygous carriers of the SNP were found to be in Hardy-Weinberg equilibrium (*p* = 0.14). In order to investigate a possible association between HRG C633T genotype and gestational hypertensive disorders, a model for regression analysis was performed. The participants were categorized in two groups consisting of heterozygous C/T combined with homozygous T/T vs homozygous C/C carriers and the appropriate analysis was carried out. First, a univariate analysis was carried out, and afterwards, a multivariate analysis after adjusting for maternal age, BMI and parity, variables with a possible association to exposure and outcome (*p* < 0.25). Odds ratios (OR) and 95% confidence intervals (CI) were calculated. Statistical significance was defined as *p* < 0.05.

## Results

### Background characteristics among cases and controls

Demographic and clinical characteristics of cases (*n* = 92) and controls (*n* = 200) are presented in Table 1. In the group of cases, 48.9% were diagnosed with preeclampsia (5% of which had developed superimposed preeclampsia), 50% with gestational hypertension and 1% with HELLP syndrome. Cases were older and had higher BMI compared with controls (Table 1). Nicotine use and parity were similar among cases and controls. Furthermore, the overall health status of cases and controls did not differ (data not shown). Among controls, 5.5% had been investigated for a bleeding episode during pregnancy, whereas none of the cases had suffered such an episode.

**Table 1:** Distribution of epidemiological data among cases and controls

	Cases	Controls	
Maternal age, yrs	32 (IQR = 7)	31 (IQR = 7)	*p* = 0.012
Maternal BMI, kg/m^2^	25.97 (IQR = 6.52)	23.55 (IQR = 5.18)	*p* < 0.001
Primiparity	50/92 (54.3%)	89/200 (44.5%)	*p* = 0.118
Nicotine use	9/89 (10.1%)	13/196 (6.6%)	*p* = 0.308
Homozygous C/C	36/92 (48.3%)	105/200 (52.5%)	*p* = 0.034
Heterozygous C/T	42/92 (39.7%)	74/200 (37%)	*p* = 0.180
Homozygous T/T	14/92 (12%)	21/200 (10.5%)	*p* = 0.249
Gestational length, weeks	39 (IQR = 2)	40 (IQR = 1)	*p* = 0.014
Male Infant Gender	50/91 (54.9%)	108/199 (54.3%)	*p* = 0.915
Birthweight, gr	3410 (IQR 896)	3680 (IQR658)	*p* = 0.001
Birthlength, cm	50 (IQR 4)	51 (IQR 3)	*p* = 0.014

The values are reported as *n/N* (%) or median ± interquartile range

### Genetic distribution among cases and controls

Regarding the genotype distribution of the HRG C633T SNP in the population, the homozygous C/C genotype was more prevalent among controls compared to cases (Table 1).

### Pregnancy outcomes among cases and controls

In relation to pregnancy outcomes, cases had shorter gestational length compared to controls and delivered infants with lower birth weight and length (Table1). Within the group of cases, it was observed that none suffered from early onset preeclampsia (defined as delivery < 34 gestational weeks), 6 women delivered prematurely (< 37 gestational weeks) and the rest (86 out of 92, 93.5%) delivered at term. In the control group, 3 women delivered during gestational week 36, whereas the rest had term deliveries (≥37 gestational weeks). No difference in relation to intrapartum or postpartum complications was observed between controls and cases.

### Background characteristics based on genotype distribution

The data were also examined after genotype stratification and no differences were noted regarding pregnancy outcomes (i.e. maternal age and BMI, nicotine use, primiparity, infant birth weight and length, gestational length and male infant gender) (Table 2).

**Table 2:** Distribution of epidemiological data among different genotypes for HRG C633T polymorphism in the entire study population

	Homozygous C/C	Heterozygous C/T	Homozygous T/T	
Genotype distribution	141/292 (48.3%)	116/292 (39.7%)	35/292 (12%)	
Maternal age, yrs	31 (IQR = 7)	32 (IQR = 6)	33 (IQR = 7)	*p* = 0.346
Maternal BMI, kg/m^2^	24 (IQR = 5.92)	24.46 (IQR = 5.58)	22.86 (IQR = 4.86)	*p* = 0.676
Primiparity	65/141 (45.5%)	54/116 (46.6%)	20/35 (57.1%)	*p* = 0.483
Nicotine use	12/139 (8.6%)	8/112 (7.1%)	2/34 (5.9%)	*p* = 0.829
Gestational length, weeks	40 (IQR = 1)	39 (IQR = 2)	40 (IQR = 1)	*p* = 0.325
Male Infant Gender	81/140 (57.9%)	57/115 (49.6%)	20/35 (57.1%)	*p* = 0.394
Birthweight, gr	3615 (IQR = 800)	3665 (IQR = 685)	3420 (IQR = 945)	*p* = 0.394
Birthlength, cm	51 (IQR = 3)	51 (IQR = 2)	50 (IQR = 4)	*p* = 0.562

The values are reported as *n/N* (%) or median ± interquartile range

Furthermore, in order to investigate the effect of the HRG C633T polymorphism on neonatal development (i.e. birth weight and length) which was due to the effect of hypertensive disorders, the analysis was carried out within the control group only (*n* = 200); no difference was noted between fetal growth (birth weight and length) and HRG C633T genotype among controls (*p* > 0.05). Moreover, no association was found in relation to genotype status and systolic or diastolic blood pressure; neither when measured in gestational week 10, nor close to delivery (data not shown).

### Logistic regression analysis

Logistic regression analysis was conducted to investigate the effect of the HRG C633T polymorphism on the development of gestational hypertensive disorders (i.e. new onset, as well as superimposed preeclampsia, gestational hypertension and HELLP syndrome). A trend towards increased risk of gestational hypertensive disorders was observed among the T allele carriers in comparison to homozygous C/C, i.e. heterozygous C/T [OR 1.66, 95% CI (0.97–2.83), *p* = 0.066], as well as homozygous T/T [OR 1.94, 95% CI (0.90–4.22), *p* = 0.093)]. After grouping the population according to their genotypic distribution, the odds for gestational hypertensive disorders were higher among heterozygous C/T or homozygous T/T [OR 1.72, 95% CI (1.04–2.84), *p =* 0.034], compared with homozygous C/C. The association remained significant even after adjustment for maternal age, maternal BMI and parity (Table 3).

**Table 3:** Regression analysis among cases/controls and genotype combinations of HRG C633T SNP

	Homozygous C/C	Heterozygous C/T or Homozygous T/T	
Gestational Hypertensive disorders (unadjusted model)	1	OR1.72 (1.04–2.84)	*p* = 0.034
Gestational Hypertensive disorders (adjusted model)	1	aOR 1.77 (1.04–3.02)	*p* = 0.036

Adjusted for maternal age (as a continuous variable), BMI (as a continuous variable) and Parity (as dichotomous variable, Primipara vs Multipara)

## Discussion

In this study we demonstrated that inheritance of the T-allele of the HRG C633T SNP (i.e. heterozygous C/T and homozygous T/T carrier status) is associated with a risk of developing gestational hypertensive disorders. Our findings are in line with previous studies demonstrating the T-minor allele (homozygous T/T genotype) to be less favourable, both regarding fertility [9, 16, 17] and the occurrence of recurrent pregnancy loss [14].

Recurrent pregnancy loss and preeclampsia most often manifest at different stages of a pregnancy, yet both originate from deficient trophoblast invasion during early gestation [20]. Between these two extreme ends, variable degrees of trophoblast invasion are compatible with on-going pregnancy, but result in inappropriate conversion of the spiral arteries and thus inadequate placentation might develop [20]. Moreover, although a predisposition to vascular dysfunction may exist pre-conceptionally, it is ultimately the disruption of the finely tuned balance between proangiogenic and antiangiogenic factors secreted by the dysfunctional placenta that converge on the vasculature that lead to this syndrome [21]. However, because hypertensive disorders in pregnancy, including preeclampsia, are a heterogeneous, polygenic and multifactorial group of disorders, no single factor, has so far been described as being responsible for the condition [22, 23]. On the contrary, multiple genes involved in various biological pathways must be deranged for the syndrome to develop [23]. It seems therefore more appropriate to claim that there are multiple candidate genes, that act as susceptibility loci, which, after complex interaction with environmental modifiers (such as obesity or smoking), enhance or lower a woman’s threshold for developing gestational hypertensive disorders.

To date, it has been shown that low levels of serum HRG during pregnancy have been associated with early-onset preeclampsia [24]. Furthermore, in preeclampsia models, VEGF (Vascular Endothelial Growth Factor) and PlGF (Placental Growth Factor) exhibit a proangiogenic role, whereas sFlt-1 (soluble form of the receptor for VEGF) acts as an antagonist of VEGF and PlGF, resulting in endothelial cell dysfunction and vasoconstrictor effects in maternal circulation [23]. Moreover, HRG peptides reflecting the C633T polymorphism added in endometrial culture models have been associated with low VEGF-induced proliferation, but increased migration of human endometrial endothelium and enhanced tube formation, leading to affected angiogenesis [16].

Proline to serine missense mutation on position 204 (corresponding to the HRG C633T SNP) allows glycosylation at position 202, which is located near a disulphide bond(Cys 185-Cys 407), entailing the proteolytic HRR fragment of the HRG protein. It has been postulated that differences in glycosylation could possibly affect protein stability and/or degradation rates and thereby affect HRG concentration and its antiangiogenic properties [25]. The exact pathophysiological mechanism of how HRG contributes to preeclampsia still remains to be discovered.

The allele frequency of the HRG C633T SNP in a European population (Hap-Map Eu) is 0.69 for the proline (C-allele) and 0.31 for the serine (T-allele) alleles, respectively [26]. In accordance, in the entire study population it was estimated that MAF = 0.32. We regard that it is a case of co-dominance inheritance pattern [27] as the disease risk in heterozygous lies between that of homozygous C/C or T/T but not in a specific way, such as the one in a multiplicative or additive model. Thus, a logistic regression model was regarded as being appropriate to examine the association [27].

### Strengths and limitations

This is, to the best of our knowledge, the first study examining the association between gestational hypertensive disorders and HRG C633T SNP carrier status. No power calculation could be made, as no similar study has been performed before. The study can therefore be regarded as a pilot study. Additionally, when the study was initiated (year 2011) genetic studies were not that common and the laboratory analysis had a higher cost which prohibited including a higher number of participants. Larger studies are thus needed to verify the results.

One of the major strengths of the study is the well-defined study population with the inclusion of accurate information, both in relation to fertility/infertility status, as well as in relation to the diagnosis of preeclampsia and other hypertensive disorders of pregnancy. The nested case-control study design, with participants primarily selected on the basis of their outcome within the same cohort (BASIC), followed prospectively starting at pregnancy week 17, is considered appropriate. The risk of recall bias and uncertainty was reduced, as all clinical and background information were collected before the onset of the disease and were retrieved from the participant’s medical record. Furthermore, because the genotype was unknown, both for the participant and the researcher, there was limited risk of selection bias.

It is known that ethnicity might affect the distribution of the polymorphism as well as the rate of preeclampsia. However, both cases and controls originate from the same geographic area, thus limiting the influence of this factor. Furthermore, despite not knowing the exact ethnic origin of the population in our study, it is estimated that Caucasians constitute 90–95% of the population living in the area [28]. Through the random selection of cases and controls from the same cohort, we have ensured a good match between the genetic background of cases and controls, which is adequate in preventing population stratification.

Because only 22% of women invited to participate in the BASIC cohort accepted, the results cannot be readily generalizable for the whole of the Uppsala population. Pregnant women with higher age, higher education and primiparas are slightly overrepresented among the BASIC study participants compared to the general population [18]. The higher educational and social status of participants might be partly explained by the underrepresentation of women with foreign background due to the language communication requirement, as well as due to the well-known fact that higher educated women have higher participation rates in studies [18]. The latter should be taken into account, as socioeconomic status may be related to the study’s main outcome. We have, however, no reason to believe that that would affect genotype distribution. It might, however, in part, reflect the lower incidence of severe or early onset preeclampsia among cases in our study. Late onset preeclampsia, occurring in > 75% of preeclamptic pregnant women [23], appears to have a weaker placental component [23] with maternal genetic, behavioural and environmental factors instead having a higher impact [29]. Nonetheless, there is still evidence of increased incidence of placental pathology and impaired vascular remodelling compared with normal pregnancy [30]. Based on the study findings, we cannot deduct any safe assumptions about early-onset preeclampsia, which is usually related to pronounced impaired angiogenesis and disturbed remodelling of the spiral arterioles [23, 30]. We could only speculate that the results from a genetic study would have been more prominent if those women who suffered severe or early-onset preeclampsia were included compared to a mixed population.

One should, however, always be cautious when interpreting the genetic effects, as a positive association is not always proof of a direct causation, but might be sign of linkage disequilibrium, population stratification or might have occurred by random chance. In vitro studies might help to clarify the biological mechanisms behind the effects of HRG C633T SNP on the development of gestational hypertensive disorders.

Although the effect of the HRG SNP studied in gestational hypertensive disorders is considered moderate, it could be included together with other associated SNPs in larger studies with more complex design, such as meta-analysis and pathway analysis [23], investigating the correlation between this relevant phenotype and genotype.

## Conclusions

Our study evaluated the relationship between HRG C633T SNP and risk of gestational hypertensive disorders and suggested that carrier status of HRG SNP might affect the occurrence of gestational hypertensive disorders. So far, no single biomarker has been found to predict the development of gestational hypertensive disorders with sufficient accuracy in order to be clinically useful [22]. The HRG C633T SNP, along with other biomarkers, could become part of an algorithm for predicting individual susceptibility for gestational hypertensive disorders in the future.

## Abbreviations

HRG: Histidine Rich Glycoprotein
PE: Preeclampsia
SNP: Single Nucleotide Polymorphism
VEGF: Vascular Endothelial Growth Factor
